# Management of Lateral Semicircular Canal Benign Paroxysmal Positional Vertigo

**DOI:** 10.3389/fneur.2020.01040

**Published:** 2020-09-15

**Authors:** Francisco Zuma e Maia, Bernardo Faria Ramos, Renato Cal, Camila Martins Brock, Pedro Luiz Mangabeira Albernaz, Michael Strupp

**Affiliations:** ^1^Department of Otorhinolaryngology and INSCER, Pontifical Catholic University of Rio Grande do Sul, Porto Alegre, Brazil; ^2^Department of Otorhinolaryngology, Federal University of Espirito Santo, Vitoria, Brazil; ^3^Department of Otorhinolaryngology, University Center of Para (CESUPA), Belem, Brazil; ^4^Department of Otorhinolaryngology, Pontifical Catholic University of Rio Grande do Sul (PUC-RS), Porto Alegre, Brazil; ^5^Department of Surgery, Hospital Israelita Albert Einstein, São Paulo, Brazil; ^6^Ludwig Maximilians University, Munich, Germany; ^7^Department of Neurology and German Center for Vertigo and Balance Munich, Munich, Germany

**Keywords:** apogeotropic nystagmus, benign paroxysmal positional vertigo, canalolithiasis, cupulolithiasis, geotropic nystagmus, horizontal semicircular canal, lateral semicircular canal, repositioning maneuvers

## Abstract

Benign paroxysmal positional vertigo (BPPV) is the most common cause of peripheral vestibular vertigo. It is caused by free-floating otoconia moving freely in one of the semicircular canals (canalolithiasis) or by otoliths adhered to the cupula (cupulolithiasis). The posterior canal is the most common canal affected, followed by the lateral canal. Diagnosis of the side affected is critical for successful treatment; therefore, suppressing visual fixation is essential to examination of these patients' eye movement. On the basis of our experience, we have adopted the Zuma maneuver and the modified Zuma maneuver for both apogeotropic and geotropic variants of lateral canal BPPV. Knowledge of the anatomy and pathophysiologic mechanisms of the semicircular canals is essential for correct management of these patients. Hence, using a single maneuver and its modification may facilitate daily neurotological practice.

## Introduction

Benign paroxysmal positional vertigo (BPPV) is the most common cause of peripheral vestibular vertigo. The lifetime prevalence, the 1-year prevalence, and the 1-year incidence of BPPV were estimated at 2.4, 1.6, and 0.6%, respectively (1). The condition is characterized by brief recurrent attacks of vertigo induced by changes in head position relative to gravity, mainly when looking up, rolling over in bed, or straightening up after bending over (2). In most patients, it is caused by free-floating otoconia moving freely in one of the semicircular canals (canalolithiasis) (3–5). More rarely, otoconia are adhered to the cupula (cupulolithiasis) (6).

The cause of BPPV is mostly idiopathic. However, there are potential risk factors associated with higher incidence of BPPV, such as advancing age (7), migraine (8), genetic predisposition (9), head trauma (10, 11), vitamin D deficiency (12–14), low solar radiation exposure (15–17), and other inner ear diseases (i.e., vestibular neuritis, Menière's disease, and sudden sensorineural hearing loss) (18–20).

The posterior semicircular canal variant of BPPV (PC-BPPV) is a well-recognized condition, since it was described in 1952. It is characterized by a torsional vertical nystagmus provoked by the Dix Hallpike maneuver (21) or diagnostic Sémont maneuver (22, 23). In contrast, the first reports of the lateral semicircular canal variant of BPPV (LC-BPPV) were published in 1985 (24, 25). This variant is characterized by linear horizontal nystagmus beating to the same side as (geotropic) or to the opposite side (apogeotropic) of the head turn in the supine roll test.

The PC is the most common canal affected, corresponding to 60 to 79% of all BPPV cases, followed by the LC, which accounts for 16 to 31% of cases (26–29). Both subtypes of BPPV can present with similar symptoms, although attacks may last longer and be more intense in LC-BPPV. Initially, the autonomic symptoms and the vertigo may be so severe, and provoked by any head or body movement, that patients sometimes only describe spontaneous and not positional vertigo (30). Mostly, however, the vertigo is provoked by lateral turning movements, leading patients with LC-BPPV to avoid turning their heads. Nevertheless, LC-BPPV has a higher rate of spontaneous resolution than PC-BPPV (25, 31). This can be understood if the spatial orientations of the semicircular canal are considered. The LC inclines upward and its cupular barrier is at the upper end. As a result, otoliths floating in the LC tend to move back to the utricle more easily (27).

## Diagnosis of LC-BPPV

BPPV is a type of episodic recurrent vertigo provoked by head movement, but it may also present as an acute vestibular syndrome. A bedside examination should therefore be included as part of the physical examination of these patients.

Diagnosis of the side affected is critical for successful treatment (32). An important clinical sign for identifying the affected side in LC-BPPV is the intensity of the nystagmus evoked by the supine head roll test or McClure-Pagnini test. This maneuver can induce horizontal nystagmus that may beat toward the ground (geotropic variant) or toward the ceiling (apogeotropic variant). The geotropic variant is attributed to free floating particles in the posterior arm of the LC. In contrast, the apogeotropic variant of LC-BPPV is attributed to free floating particles in the anterior arm of the LC, particles attached to the cupula facing the canal, or particles attached to the cupula facing the utricle (33–36).

The McClure-Pagnini test is performed by turning the head about 90° to each side in a supine position. Since it is performed on the yaw plane, it should be more correctly called the head yaw test (HYT) while supine (30, 32, 37). The nystagmus beats with greater intensity toward the affected ear, according to Ewald's second law, which postulates that the response to an excitatory stimulus is always more intense than to an inhibitory stimulus. In geotropic LC-BPPV, the otoliths will move toward the ampulla during the HYT toward the affected ear, resulting in an ampullopetal excitatory current and causing a nystagmus beating toward the affected ear. Turning the head to the unaffected side, the particles will move away from the ampulla, resulting in an ampullofugal inhibitory endolymphatic current, causing a nystagmus beating to the unaffected ear. Conversely, in apogeotropic LC-BPPV (30, 38), the particles will move away from the ampulla during the HYT to the affected ear, resulting in an ampullofugal inhibitory endolymphatic current, causing a nystagmus beating toward the unaffected ear. Turning the head to the healthy side, the particles will move toward the ampulla, resulting in an ampullopetal excitatory endolymphatic current, causing a nystagmus beating toward the affected ear. Hence, in apogeotropic LC-BPPV, the affected side is the side on which the nystagmus is less intense.

However, sometimes it may be difficult to identify the differences in intensity of nystagmus in the HYT. As a result, several tests have been described for secondary signs of lateralization for identification of the affected side (39, 40).

In the Seated Supine Positioning Test (SSPT) (41, 42), the patient is briskly brought from a seated to the supine position. When the patient is brought to the supine position with the head flexed at 30°, the LC is on a vertical plane and the particles are pushed downwards. In geotropic LC-BPPV, in which the otoliths are located in the posterior arm of the LC, they move toward the utricle and away from the ampulla. This results in an ampullofugal inhibitory endolymphatic current and causes a nystagmus beating toward the unaffected ear. In apogeotropic LC-BPPV, in which the otoliths are in the anterior arm of the LC or adhered to the cupula, they move toward the ampulla. This results in an ampullopetal excitatory endolymphatic current and therefore the nystagmus beats toward the affected side.

The bow and lean test was described in 2006 (40). Since it is performed on the pitch plane, it should be more correctly called the head pitch test (HPT). First, it is necessary to confirm whether the type of LC-BPPV is a geotropic or apogeotropic variant, using the HYT. Next, the direction of nystagmus is noted when the patient bows the head over 90° and leans the head backward over 45° in the sitting position. In geotropic LC-BPPV, the otoliths move toward the ampulla in the bow test and away from the ampulla in the lean test. In contrast, in apogeotropic LC-BPPV, the otoliths move away from the ampulla in the bow test and toward the ampulla in the lean test.

Evaluation of nystagmus intensity and direction during the HPT can also be useful to distinguish between the geotropic and apogeotropic variants and to identify the affected side (39). According to a previous study, nystagmus with greater intensity in the bow test than the lean test indicates an ampullopetal excitatory endolymphatic current and suggests a geotropic LC-BPPV affecting the same side as the direction of the nystagmus. Hence, if there is an intense nystagmus beating to the right in the bow test, the particles are located in the posterior arm of the right LC (geotropic LC-BPPV). Conversely, a nystagmus with greater intensity in the lean test than the bow test indicates an ampullofugal inhibitory endolymphatic current and suggests an apogeotropic LC-BPPV affecting the same side as the direction of the nystagmus.

Patients with LC-BPPV may also exhibit a pseudo-spontaneous nystagmus (PSN) and this can be differentiated from spontaneous nystagmus with the HPT in the sitting position (32). In the HPT, PSN increases its intensity with head extension over 30° and reverses direction with the head bent over 60°. The nystagmus may also stop when the head is bent to 30° (neutral position), since the LC is aligned in respect to the horizontal plane in this position. This nystagmus can be provoked by slow rotation of the patient's head horizontally, since this maneuver raises the percentage of patients who exhibit PSN to 96% (32). Inclination of the LC in respect to the horizontal plane allows the otoliths to move under the action of gravity. In geotropic LC-BPPV, the particles flow away from the ampulla and cause a nystagmus beating toward the unaffected ear. On the other hand, in apogeotropic LC-BPPV, the otoliths are pushed toward the ampulla and therefore the nystagmus beats toward the affected ear.

On the basis of our experience, we have adopted the strategy of the minimum stimulus for diagnosis of LC-BPPV (41). First, we rotate the patient's head slowly in the horizontal plane and check whether there is PSN. Then we perform the HPT, and check whether there is a horizontal nystagmus that changes direction with this test. If this nystagmus is observed, we proceed with the SSPT followed by the HYT.

## Management of the Apogeotropic Variant of Lateral Canal Benign Paroxysmal Positional Vertigo (Apogeotropic LC-BPPV)

Choosing the correct repositioning procedure for the treatment of LC-BPPV is very complicated, since diagnosis of the affected side and the subtype of the BPPV is critical for successful treatment (43).

Apogeotropic LC-BPPV is attributed to particles attached to the cupula facing the canal, particles attached to the cupula facing the utricle, or free-floating particles in the anterior arm of the LC (33–36). Consequently, the objective of the repositioning maneuver for this variant is to detach the otoliths from the cupula (in patients with cupulolithiasis facing the canal) and remove them and free particles from the anterior arm through the posterior arm toward the utricle. Otoliths that are adhered to the cupula on the utricular side can move straight to the utricle during the repositioning maneuver.

Several repositioning treatments have been proposed for apogeotropic LC-BPPV, such as the new Gufoni maneuver for the apogeotropic form of LC-BPPV (44, 45), head-shaking in the horizontal plane (46, 47), the modified Sémont maneuver (47, 48), the cupulolith repositioning maneuver (CuRM) (49) and, recently, the Zuma maneuver (50). Table 1 lists the pros and cons linked to each of these maneuvers for apogeotropic LC-BPPV.

**Table 1:** Pros and cons of repositioning maneuvers for apogeotropic LC-BPPV.

**Maneuver**	**Pros**	**Cons**
Gufoni's new maneuver for apogeotropic LC-BPPV	- Transforms an apogeotropic variant into a geotropic variant - Starts by lying onto the affected side - Brisk deceleration	- Low rate of resolution with a single maneuver - Needs another maneuver after conversion to a geotropic variant - Lacks the forward head tilt before sitting up - Does not treat cupulolithiasis on the utricular side
Head-shaking in the horizontal plane	- Facilitates detachment of otoconia in cupulolithiasis - Can be combined with other maneuvers	- Low rate of resolution with a single maneuver - Needs to be repeated at least 3 times and at home - Lacks the forward head tilt before sitting up
Modified Semont maneuver	- Starts by lying onto the affected side - Brisk deceleration	- Low rate of resolution with a single maneuver - Lacks the forward head tilt before sitting up - Does not treat cupulolithiasis on the utricular side
Cupulolith repositioning maneuver (CuRM)	- Starts by lying onto the affected side - Combined with mastoid oscillation, which can help to detach the otoconia from the cupula - Treats cupulolithiasis on the utricular side	- Low rate of resolution with a single maneuver - Lacks brisk deceleration - Lacks the forward head tilt before sitting up
Zuma maneuver	- High rate of resolution with a single maneuver - Brisk deceleration - Starts by lying onto the affected side - Forward head tilt before sitting up	- -Long lasting (3 min in each step)

**Figure 1:**
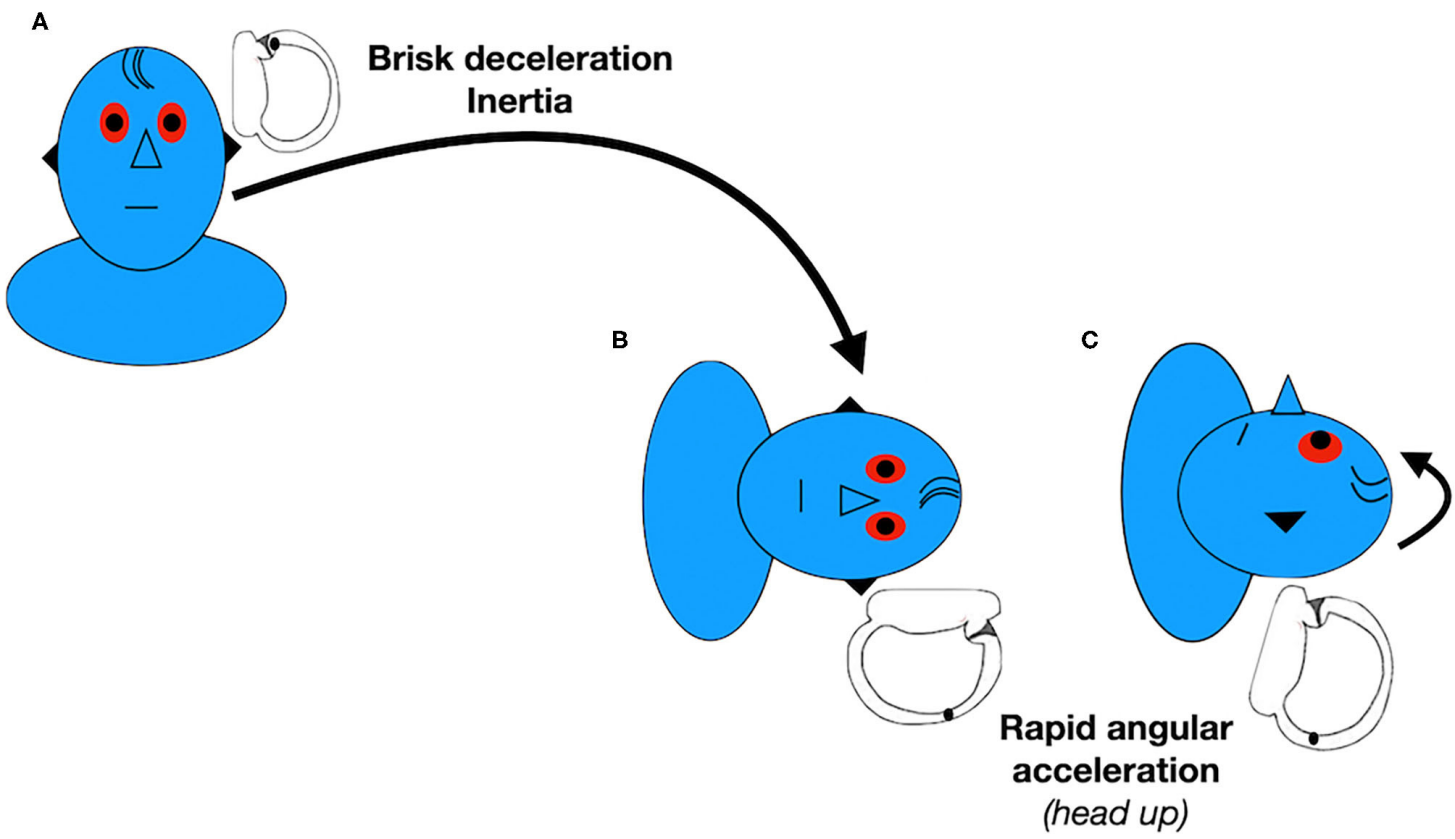
Gufoni's new maneuver for left apogeotropic LC-BPPV. Data modified from Ciniglio Appiani et al. (44).

The new Gufoni maneuver for apogeotropic LC-BPPV consists of quickly moving the patient, starting from the sitting positioning, onto the affected side, followed by a quick 45° upward turn, before returning to the sitting position (44) (Figure 1). The inertia provoked by the brisk deceleration before the patient is brought to the lying down position may detach the particles from the cupula. In this position, the anterior and posterior arm of the LC are placed in the vertical plane, so otoliths may flow from the canal side of the cupula or from the anterior arm into the posterior arm. The 45° upward head turning is intended to facilitate movement of the detached particles from the utricular side of the cupula toward the utricle or movement of the otoconia from the canal side of the cupula toward the posterior arm of the LC (51). Some authors have previously reported achieving conversion from the apogeotropic into a geotropic variant of LC-BPPV in all patients (44). A randomized clinical trial (45) observed 59% of vertigo and nystagmus resolution with a single administration of the new Gufoni maneuver for apogeotropic LC-BPPV.

Previously, authors have presented head-shaking in the horizontal plane as a treatment for apogeotropic LC-BPPV. This maneuver is intended to break otoconial debris into pieces and detach the particles from the cupula through alternate accelerating and decelerating forces (46, 47, 51). There are several descriptions of this method. According to one previous study, (46) 3 series of 30 rapid right-left shakes of the head around the yaw axis were performed with the patient in supine position and then repeated at home twice a day for at least 3 days. A more recent study (47) proposed movement of the patient's head sideways in a sinusoidal fashion at an approximate rate of 3 Hz for 15 seconds in the sitting position with the head pitched at 30°. They reported response rates of 17 and 33%, respectively. A previous randomized clinical trial (45) showed better response in patients treated with the head-shaking maneuver compared with patients who underwent a sham maneuver. However, there was no difference in comparison with patients treated with the new Gufoni maneuver for apogeotropic LC-BPPV.

**Figure 2:**
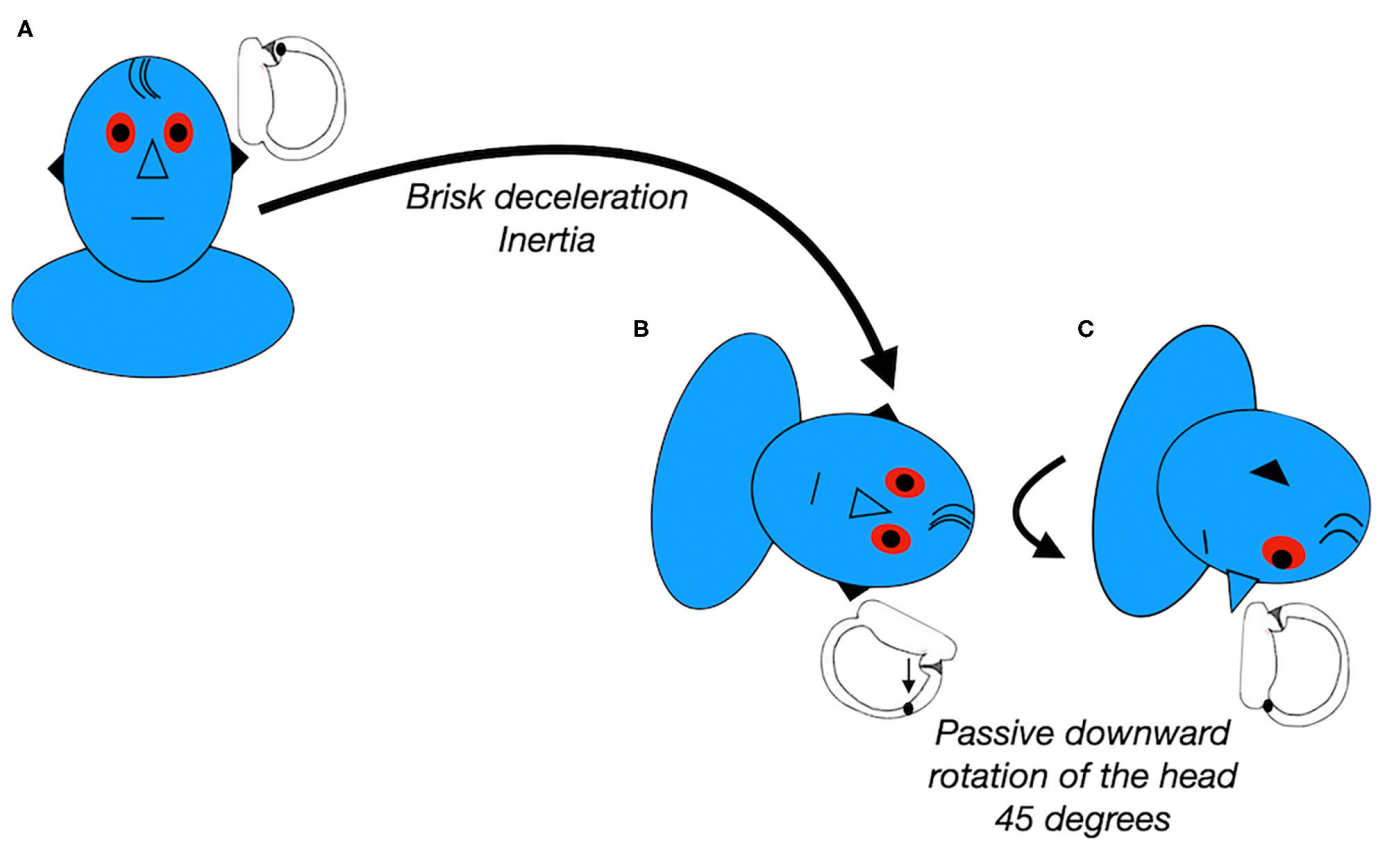
Modified Semont maneuver for left apogeotropic LC-BPPV. Data modified from Casani et al. (48).

In the modified Sémont maneuver, the seated patient is briskly brought into a side-lying positioning onto the affected side, followed by turning the head 45° downward, before returning to the sitting position (Figure 2). Theoretically, the principles of this maneuver should combine the effect of inertial and gravitational forces in order to detach the otoconia from the cupula and move it into the utricle. The efficacy of this maneuver for patients with apogeotropic LC-BPPV varies widely, ranging from 13 to 44% (47, 48).

**Figure 3:**
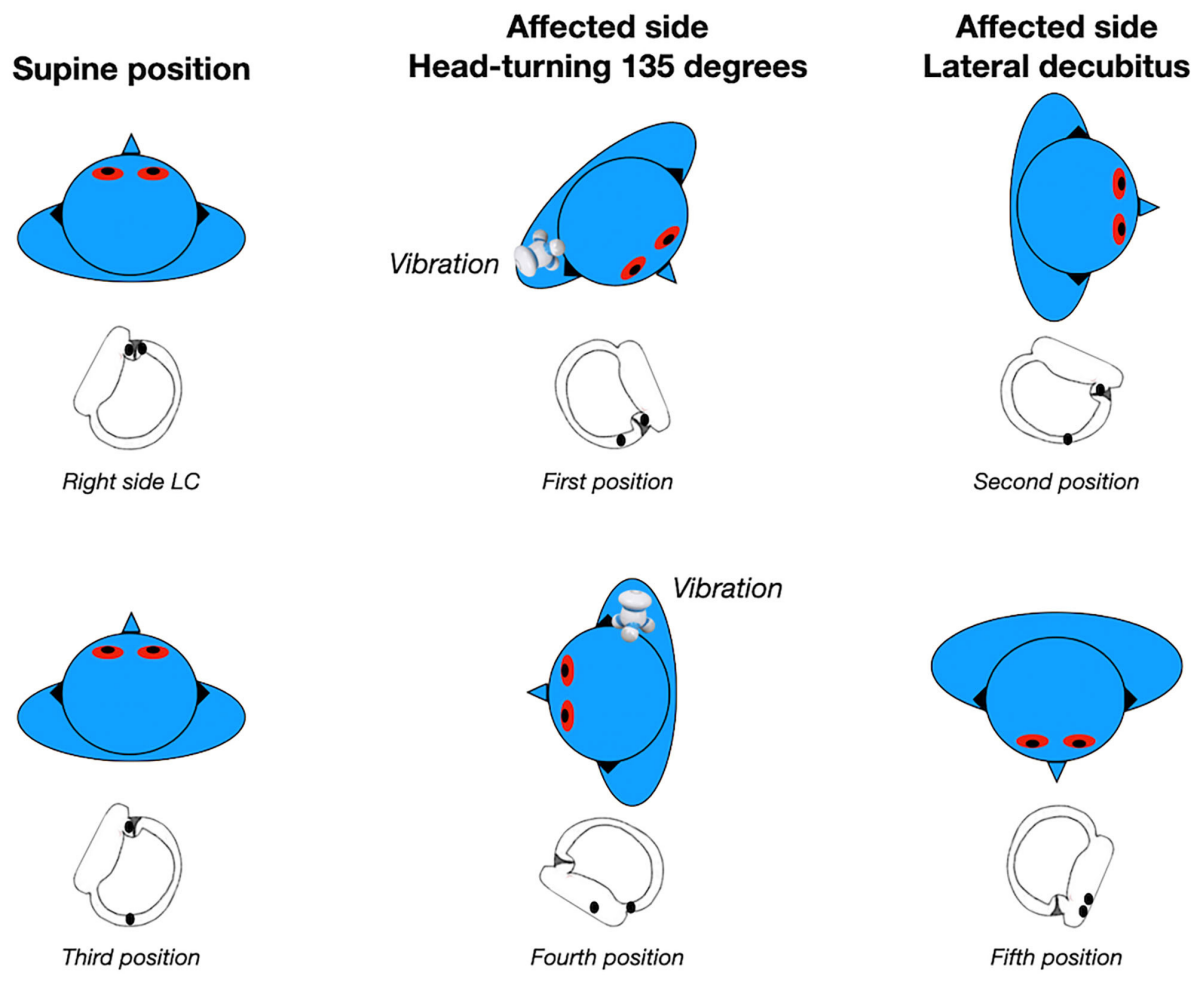
Cupulolith repositioning maneuver (CuRM) for right apogeotropic LC-BPPV. Data modified from Kim et al. (49).

The cupulolith repositioning maneuver (CuRM) (49) also aims to target cupulolithiasis in which the otoconia are facing the utricle. These authors proposed a modification of the roll maneuver with an additional step, in which the patient completes a 90° head turn to the healthy side while in supine position (51). First, the patient's head is rotated 135° to the affected side and mastoid oscillation is applied to the affected side for 30 s with a 60 Hz hand-held vibrator (1st position). Next, the patient's head is turned 45° to the healthy side (2nd position, lateral decubitus on the affected side). Then, the patient's head is turned 90° to the healthy side (3rd position, supine position). For the 4th position, the patient's head is turned 90° to the healthy side and oscillation is applied again (4th position, lateral decubitus on the healthy side). For the 5th position, the patient's head is rotated 90° in the same direction (5th position, prone position), and the patient is slowly brought back to the sitting position without neck extension (Figure 3). The mechanisms involved are a combination of mastoid oscillation for detaching the otoliths from the cupula and gravitational forces for moving them through the canal toward the utricle. A double-blind randomized prospective study did not detect statistically different therapeutic efficacy comparing the CuRM (38%) with the head shaking maneuver (12%) (52). However, the resolution rate with the head-shaking maneuver was very low in this study.

**Figure 4:**
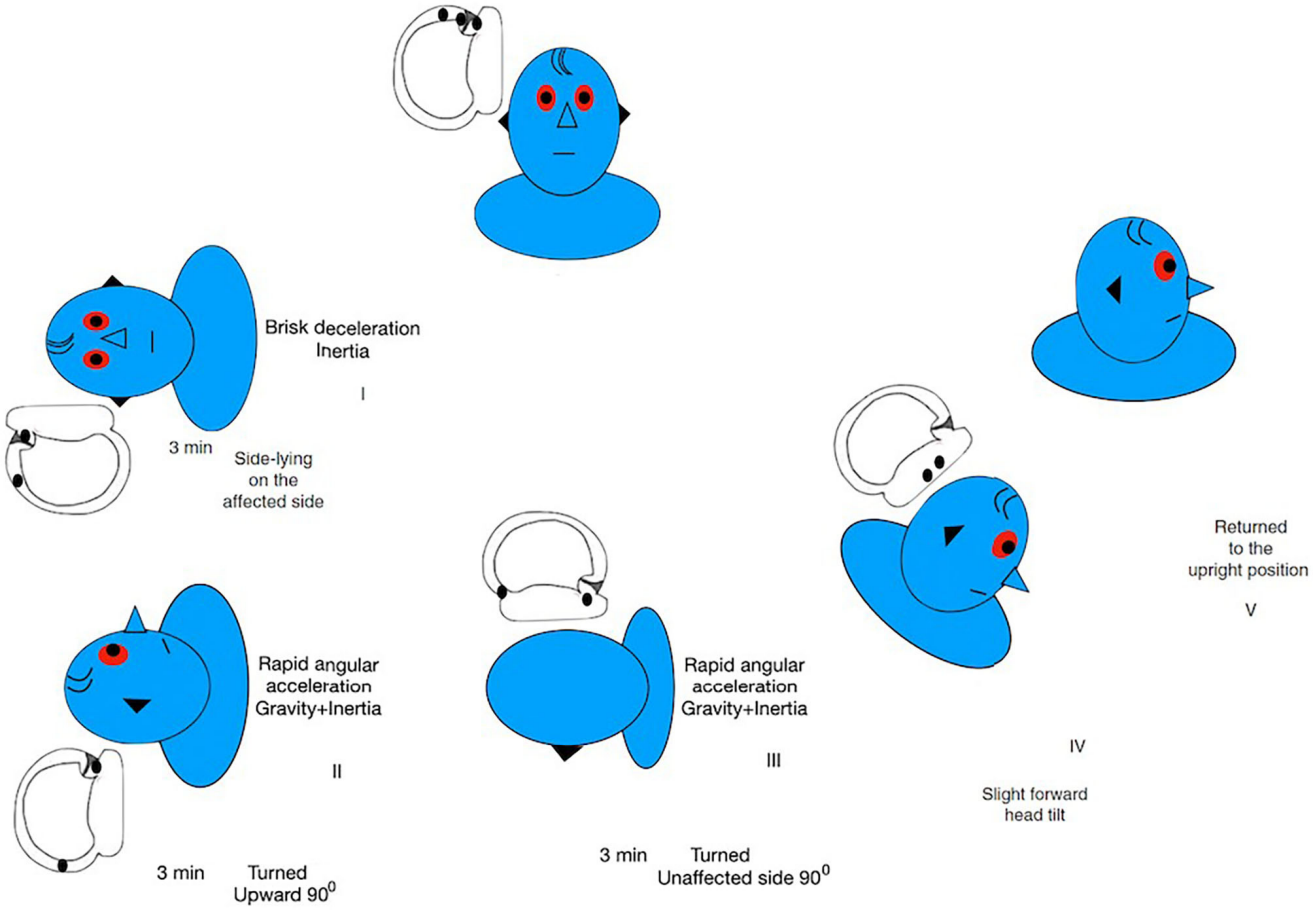
Zuma Maneuver for right apogeotropic LC-BPPV. Data modified from Ramos et al. (36) and Zuma e Maia (50).

The Zuma maneuver (50) was proposed in 2016 for detaching both the otoconial debris from the anterior arm of the semicircular canal and the debris attached to the cupula. It is performed with the patient in the sitting position. First, the patient is asked to quickly lie down on the affected side (step I) and is held in this position for 3 min. Then, the patient's head is rotated 90° toward the ceiling (step II) and held in this position for another 3 min. After 3 min, the patient moves the body into dorsal decubitus and the head is turned 90° toward the unaffected side (step III) and held in this position for another 3 min. Finally, the patient's head is tilted slightly forward (step IV), followed by a slow return of the patient to the sitting position (step V) (Figure 4). The forward head tilt before sitting up in step IV was proposed to avoid enabling the particles to move back toward the posterior arm of the canal. This maneuver was highly effective in a study with 8 patients with administration of a single maneuver (50). It combines the inertial and gravitational forces to both detach the otoliths and move them toward the utricle. A recent retrospective study (53) compared patients treated with the Zuma maneuver or the modified Gufoni maneuver for apogeotropic LC-BPPV. It reported rates of vertigo and nystagmus resolution in patients with no previous history of BPPV of 59% and 48% for the Zuma maneuver and the modified Gufoni maneuver, respectively. This difference was not statistically significant. However, in patients with previous episodes of BPPV, resolution rates for vertigo and nystagmus were, respectively, 82 and 64% for the Zuma maneuver and 25 and 13% for the new Gufoni maneuver.

Furthermore, we have previously demonstrated the usefulness of observing the pattern of the nystagmus evoked in each step of the Zuma maneuver in patients with apogeotropic LC-BPPV (36). According to the hypothesis presented previously, we can deduce where otoliths are located. We can also elucidate the otoliths' paths toward the utricle and confirm the correct diagnosis.

## Management of the Geotropic Variant of Lateral Canal Benign Paroxysmal Positional Vertigo (Geotropic LC-BPPV)

Geotropic LC-BPPV is attributed to free floating particles in the posterior arm of the LC. Consequently, the objective of the repositioning maneuver for this variant is to move the otoliths through the posterior arm into the utricle.

**Figure 5:**
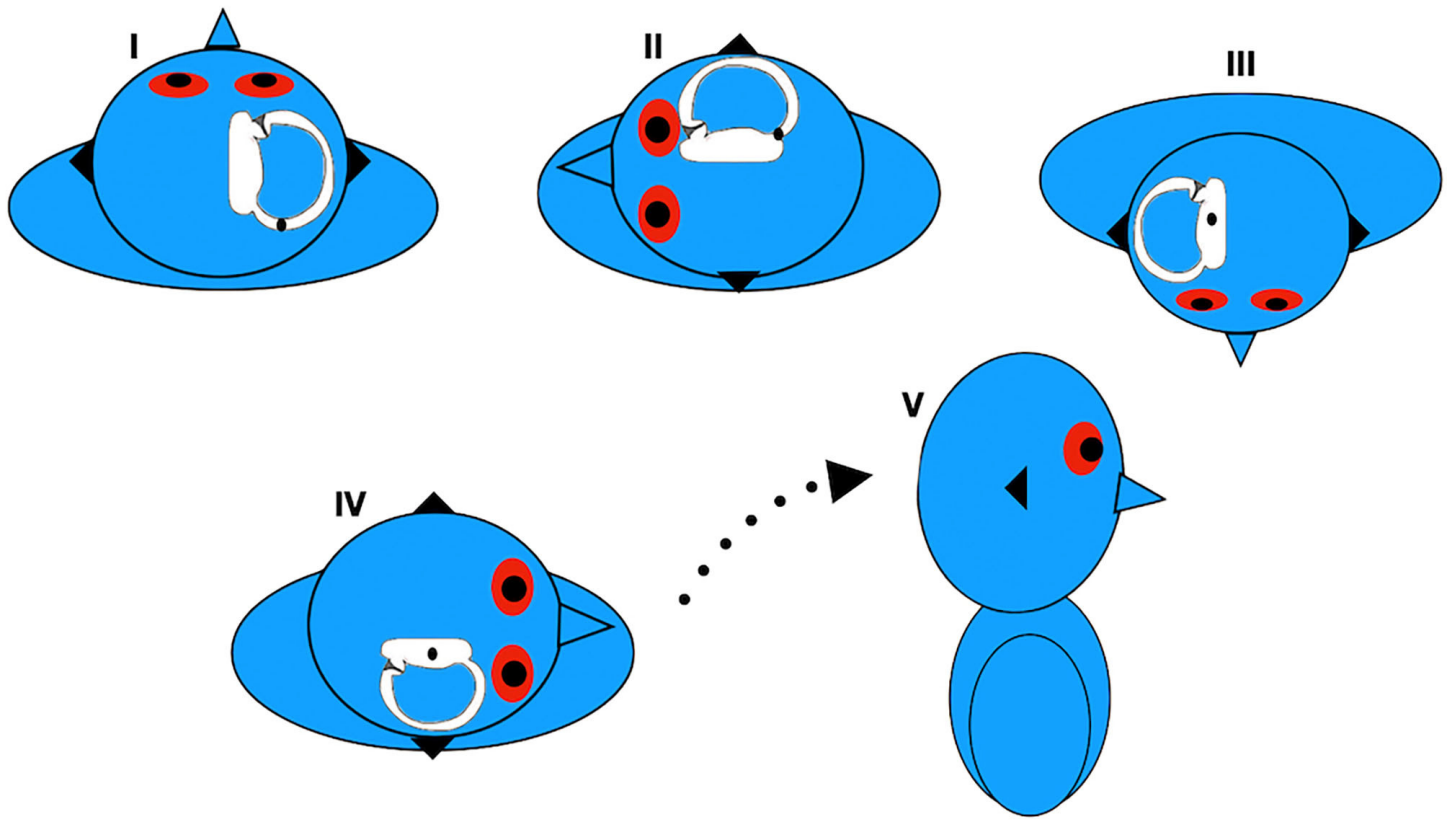
Roll maneuver for right geotropic LC-BPPV. Data modified from Lempert and Tiel-Wilck (55).

In 1994, the roll maneuver was reported for treatment of geotropic LC-BPPV. This maneuver is performed in the supine position and consists of a 270° head rotation toward the unaffected side in rapid steps of 90° at 30-s intervals (54, 55). In the same year, a modification of this maneuver was described that included a head rotation of 360° in quick steps of 90° with 60-second intervals (56) (Figure 5). Theoretically, the principles of this maneuver should combine the effects of inertial and gravitational forces, in order to move the otoconia into the utricle (51, 55, 57–59). Due to the whole-body rotation, it may be hard to perform it in patients with obesity, advanced age, or restricted cervical movement. Furthermore, these factors can affect maintenance of the head in the correct plane and the speed of the rotation (59).

**Figure 6:**
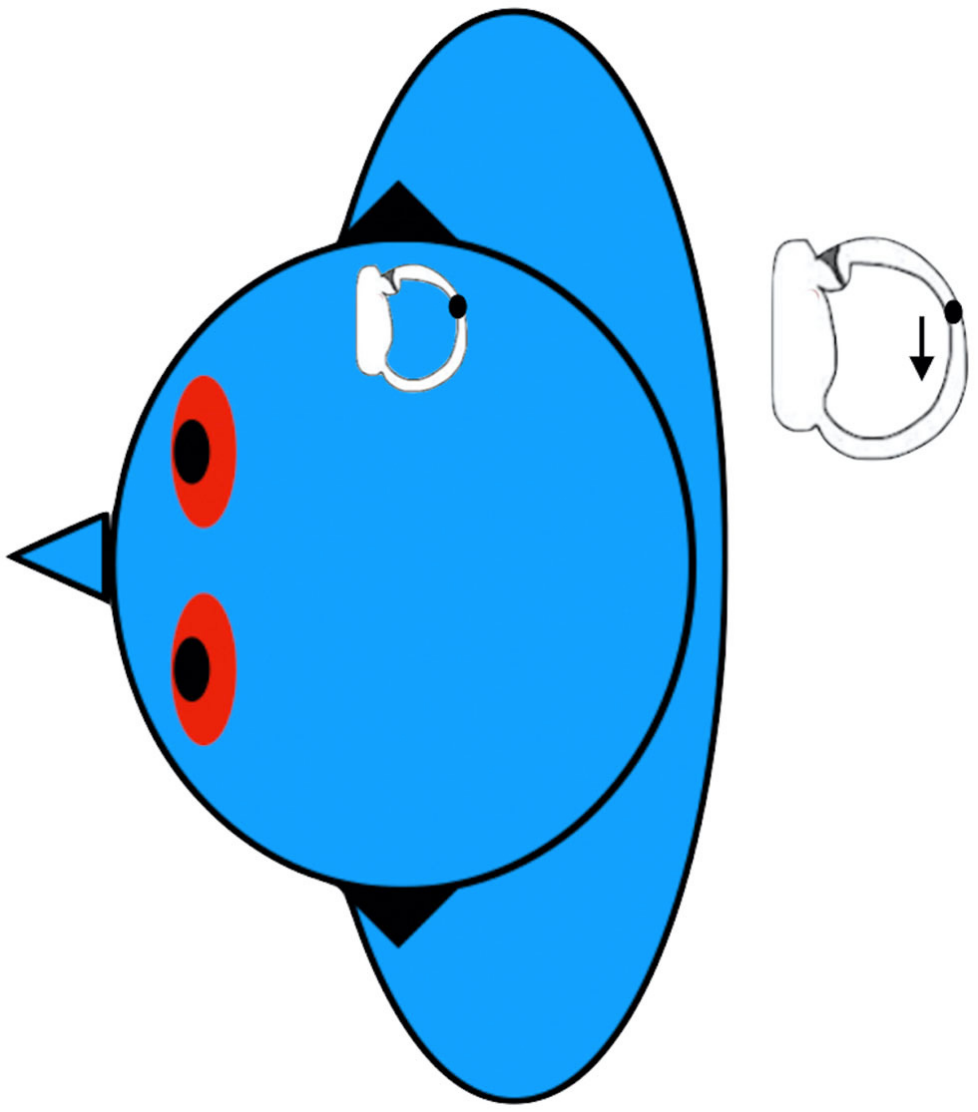
Forced Prolonged Position for right geotropic LC-BPPV. Data modified from Vannucchi et al. (46).

The Forced Prolonged Position technique was also reported in 1994 as a treatment for the geotropic variant of LC-BPPV (46, 60). Patients were asked to lie on their beds and turn their heads or whole body from the supine position toward the unaffected side (Figure 6). This position should be maintained for 12 h in order to facilitate gravitational movement of the otoliths from the posterior arm of the LC toward the utricle (57, 61, 62). However, elderly patients and patients with musculoskeletal or cardiological diseases may not manage to perform it properly (59).

**Figure 7:**
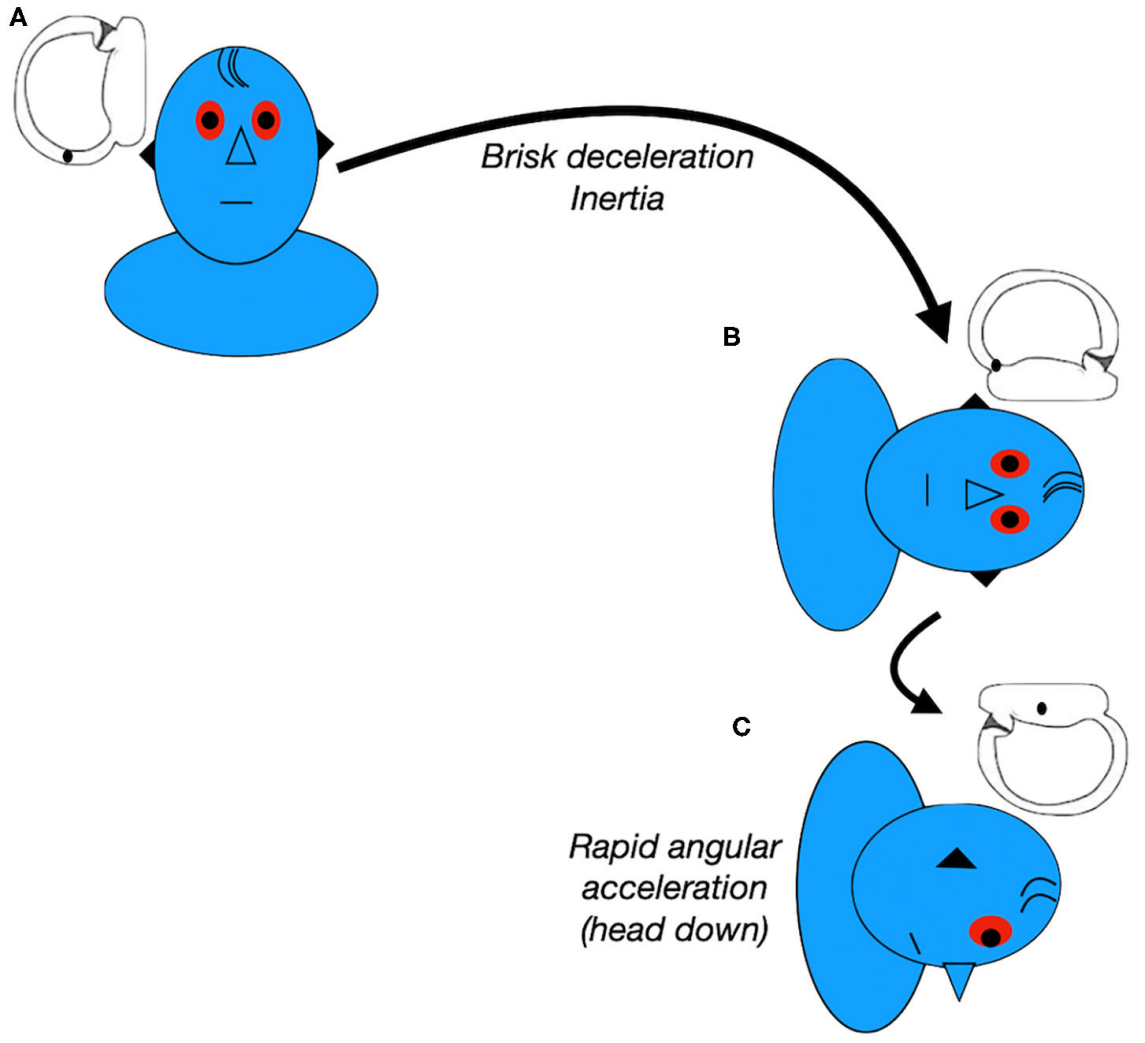
Gufoni maneuver for right geotropic LC-BPPV. Data modified from Ciniglio Appiani et al. (65).

The Gufoni maneuver was first presented in 1998 (original publication in English by Ciniglio Appiani et al. in 2001) (63–65). In this case, the patient in the sitting position is briskly moved into a side-lying position onto the unaffected side and remains in this position for 1 min after the end of the nystagmus. Then, the patient's head is quickly turned 45° downward and held in this position for 2 min. At the end, the patient slowly returns to the sitting position (Figure 7). In the side-lying position, the posterior arm of the LC is placed in the vertical plane and otoliths flow toward its nonampullated end. Since this maneuver is performed onto the unaffected side, it may be associated with less intense vertigo. The 45° downward head turning places the outlet of the posterior arm of the canal in a vertical plane and consequently facilitates movement of the particles into the utricle (58, 59).

All of these authors reported good results for treatment of geotropic LC-BPPV. The high rate of spontaneous resolution of LC-BPPV and the proximity of the posterior arm of the LC to the utricle may help with the effectiveness of repositioning maneuvers. Previous cohort studies and case series reported efficacy ranging from 67 to 100% after the Lempert maneuver (48, 55, 66, 67). Some randomized controlled studies were published recently. One of these studies found response rates of 88% after the Gufoni maneuver, compared with the sham maneuver for geotropic LC-BPPV (68). Other authors (69) showed better responses after a maximum of 2 maneuvers (Roll maneuver or Gufoni maneuver) than a sham maneuver on the initial visit day (69, 60, and 35% respectively). On the other hand, another randomized prospective clinical trial (70) compared the effectiveness of the roll maneuver plus forced prolonged positioning vs. Gufoni maneuver for geotropic LC-BPPV with response rates of 81 vs. 93%.

Table 2 demonstrates the pros and cons linked to each of these maneuvers for geotropic LC-BPPV.

**Table 2:** Pros and cons of repositioning maneuvers for geotropic LC-BPPV.

**Maneuver**	**Pros**	**Cons**
Roll maneuver	- High rate of resolution with a single maneuver - Goes from the affected to the unaffected side	- May be hard to perform in patients with obesity, advanced age, or restricted cervical movement - Lacks the forward head tilt before sitting up
Forced Prolonged Position	- May be associated with less intense vertigo	- Starts by lying onto the unaffected side - Needs to stay in this position for 12 h - Lacks the forward head tilt before sitting up - May not be performed properly by elderly patients and patients with musculoskeletal or cardiologic diseases
Gufoni maneuver	- High rate of resolution with a single maneuver - May be associated with less intense vertigo	- Starts by lying onto the unaffected side - Lacks the forward head tilt before sitting up
Zuma modified maneuver	- High rate of resolution with a single maneuver - Goes from the affected to the unaffected side	- Many steps compared to other maneuvers for geotropic LC-BPPV

Knowledge of the anatomy and pathophysiological mechanisms of the semicircular canals is essential for the correct diagnosis and treatment of any BPPV. Adhering to the concept that repositioning of otoliths should be performed from the affected side toward the healthy side, similarly to every PC-BPPV maneuver (i.e., Epley and Sémont Maneuvers), we have chosen the modified Zuma maneuver (71) for treatment of geotropic LC-BPPV. This maneuver was effective for geotropic HC-BPPV after a single application.

**Figure 8:**
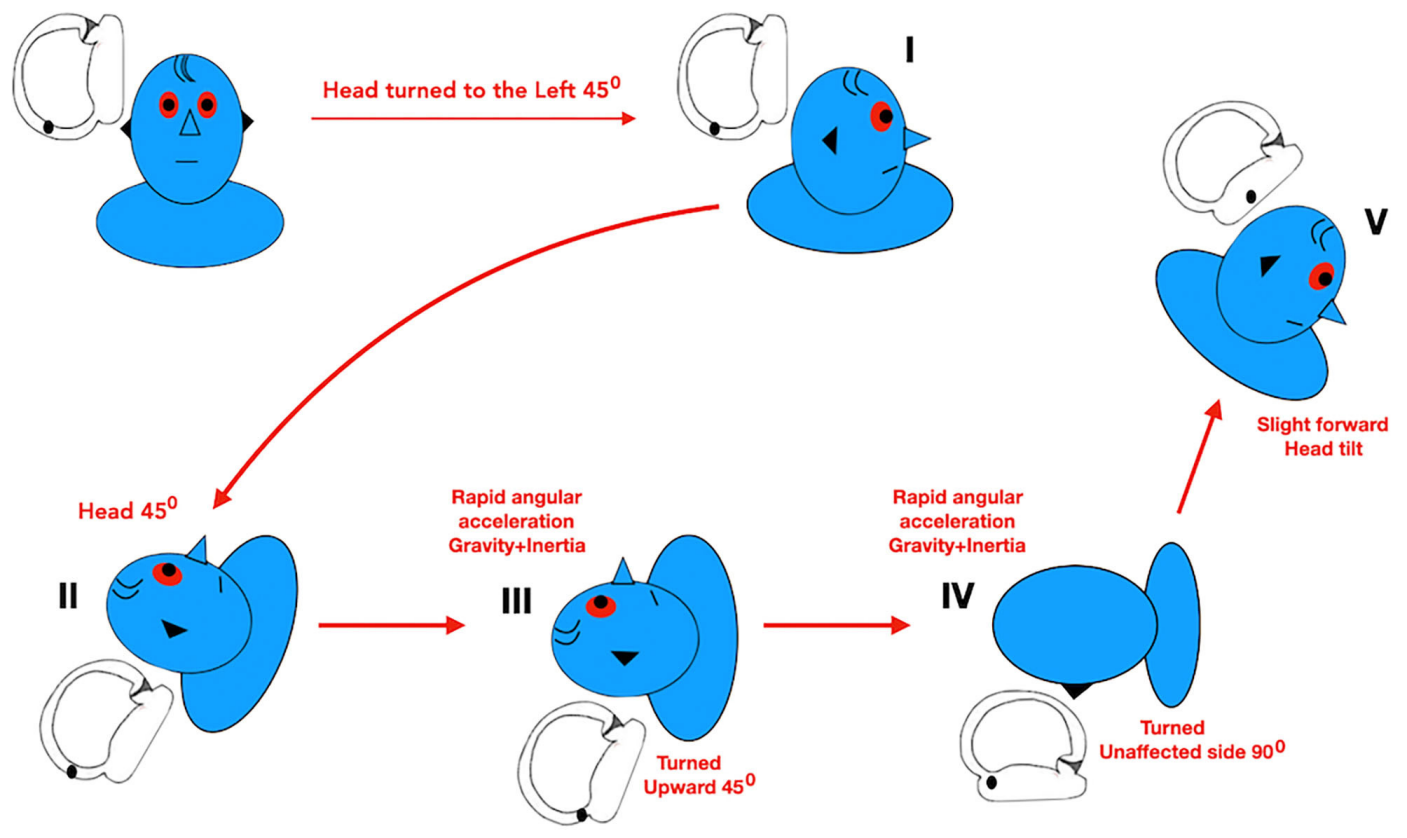
Modified Zuma maneuver for right geotropic LC-BPPV. Data modified from Ramos et al. (71).

The modification in relation to the original maneuver (50) is a 45° head turn to the unaffected side in the sitting position (step I). The patient is then asked to lie down on the affected side (step II). Next, the patient moves into dorsal decubitus and the head is turned 45° toward the unaffected side (step III). The head is then turned 90° toward the unaffected side (step IV). Finally, the patient's head is tilted slightly forward, followed by a slow return to the sitting position (step V) (Figure 8) (71).

## Conclusions

Precise diagnosis of the BPPV, the side affected, and the subtype are critical for successful treatment. We have adopted the minimum stimulus strategy (41) for evaluation of patients with suspected BPPV. Therefore, suppressing visual fixation [using Frenzel goggles, M-glasses (72), or video-Frenzel] is essential to examination of these patients' eye movement.

There is no single correct maneuver for each kind of BPPV, since several authors have reported good results with different types of repositioning maneuver. Personal experience is really important for defining a strategy to manage these patients. On the basis of our experience, we have adopted the Zuma maneuver and the modified Zuma maneuver for both apogeotropic and geotropic variants of LC-BPPV (50, 71). Knowledge of the anatomy and pathophysiologic mechanisms of the semicircular canals is essential for correct management of these patients. Hence, using a single maneuver and its modification may facilitate daily neurotological practice. Meanwhile, we can adhere to the concept that otolith repositioning should be performed from the affected side toward the healthy side.

Theoretically, based on a 3D biomechanical model of the semicircular canals (73, 74), the original Zuma maneuver could also be performed for patients with geotropic LC-BPPV. In step I of this maneuver, the otoliths, initially located in the posterior arm of the LC, flow in the direction of the anterior arm (moving away from the utricle and toward the ampulla) and cause an ampullopetal excitatory endolymphatic current. During the remaining steps of the maneuver, the otoliths would flow back to the posterior arm before entering the utricle. Therefore, for geotropic LC-BPPV, performing the modified Zuma maneuver instead of the original maneuver avoids an unnecessary excitatory stimulus and movement of the otoliths away from the utricle. In the modified Zuma maneuver, the particles only move toward the utricle, causing an inhibitory stimulus.

Another important consideration should be mentioned. In the last step of both the Zuma maneuver and the modified Zuma maneuver, before the patient returns to the sitting position, the head can be tilted slightly forward in order to encourage the particles to move toward the utricle, otherwise the otoliths could move back toward the lumen of the LC (50).

## Author Contributions

FZ, BR, RC, CB, and PM contributed to conception and design of the study. BR and FZ wrote the first draft of the manuscript. MS revised the manuscript and added suggestions about figures and tables, and the objectives of this paper. All authors contributed to manuscript revision, read, and approved the submitted version.

## Conflict of Interest

RC is a paid speaker of Grunenthal, Abbott and UCB Pharmaceutical. He received free devices for testing from Natus and Interacoustic. MS is Joint Chief Editor of the Journal of Neurology, Editor in Chief of Frontiers of Neuro-otology and Section Editor of F1000. He has received speaker's honoraria from Abbott, Actelion, Auris Medical, Biogen, Eisai, Grünenthal, GSK, Henning Pharma, Interacoustics, Merck, MSD, Otometrics, Pierre-Fabre, TEVA, UCB. He is a shareholder of IntraBio. He acts as a consultant for Abbott, Actelion, Auris Medical, Heel, IntraBio and Sensorion. He distributes the M-glasses. The remaining authors declare that the research was conducted in the absence of any commercial or financial relationships that could be construed as a potential conflict of interest.
